# Implementation of infection control best practice in intensive care units throughout Europe: a mixed-method evaluation study

**DOI:** 10.1186/1748-5908-8-24

**Published:** 2013-02-19

**Authors:** Hugo Sax, Lauren Clack, Sylvie Touveneau, Fabricio da Liberdade Jantarada, Didier Pittet, Walter Zingg

**Affiliations:** 1 Infection Control Programme, University of Geneva Hospitals and Medical Faculty, World Health Organization Collaborating Centre on Patient Safety, Geneva, Switzerland; 2 Division of Infectious Diseases and Hospital Epidemiology, University and University Hospital of Zurich, Zürich, Switzerland

**Keywords:** Implementation, Infection control, Catheter-related bloodstream infections, Hand hygiene, Intensive care units, Best practice, Organizational culture, Organizational case studies, Organizational innovation, Organizational decision making, Patient safety

## Abstract

**Background:**

The implementation of evidence-based infection control practices is essential, yet challenging for healthcare institutions worldwide. Although acknowledged that implementation success varies with contextual factors, little is known regarding the most critical specific conditions within the complex cultural milieu of varying economic, political, and healthcare systems. Given the increasing reliance on unified global schemes to improve patient safety and healthcare effectiveness, research on this topic is needed and timely. The ‘InDepth’ work package of the European FP7 **Pr**evention **o**f **H**ospital **I**nfections **b**y **I**ntervention and **T**raining (PROHIBIT) consortium aims to assess barriers and facilitators to the successful implementation of catheter-related bloodstream infection (CRBSI) prevention in intensive care units (ICU) across several European countries.

**Methods:**

We use a qualitative case study approach in the ICUs of six purposefully selected acute care hospitals among the 15 participants in the PROHIBIT CRBSI intervention study. For sensitizing schemes we apply the theory of diffusion of innovation, published implementation frameworks, sensemaking, and new institutionalism. We conduct interviews with hospital health providers/agents at different organizational levels and ethnographic observations, and conduct rich artifact collection, and photography during two rounds of on-site visits, once before and once one year into the intervention. Data analysis is based on grounded theory. Given the challenge of different languages and cultures, we enlist the help of local interpreters, allot two days for site visits, and perform triangulation across multiple data sources.

Qualitative measures of implementation success will consider the longitudinal interaction between the initiative and the institutional context. Quantitative outcomes on catheter-related bloodstream infections and performance indicators from another work package of the consortium will produce a final mixed-methods report.

**Conclusion:**

A mixed-methods study of this scale with longitudinal follow-up is unique in the field of infection control. It highlights the ‘Why’ and ‘How’ of best practice implementation, revealing key factors that determine success of a uniform intervention in the context of several varying cultural, economic, political, and medical systems across Europe. These new insights will guide future implementation of more tailored and hence more successful infection control programs.

**Trial registration:**

Trial number: PROHIBIT-241928 (FP7 reference number)

## Background

### Importance of healthcare-associated infections

Healthcare-associated infections (HAI) represent the most frequent adverse event affecting hospitalized patients, resulting in increased morbidity and mortality, longer hospital stay, and disability [1]. In the European Union, the annual number of HAIs can be estimated at approximately 4.5 million, with approximately 37,000 deaths as a direct consequence, and 16 million extra-days of hospital stay per year [2]. The risk of acquiring HAI is especially significant in intensive care units (ICU), where the World Health Organization (WHO) estimates that approximately 30% of patients are affected by one or more episodes of HAI with associated morbidity and mortality [3]. Catheter-related bloodstream infections (CRBSI) are among the leading HAI, together with urinary tract infections, surgical site infections, and ventilator-associated pneumonia [4]. CRBSI are both prototypical of the causes and preventive mechanisms of HAI, and are more distinctly defined than other HAIs [5]. Moreover, CRBSI rates are widely and consistently available among European hospitals [6].

### Prevention of vascular catheter-related bloodstream infection

Evidence indicates that CRBSI rates are amenable to substantial reduction by the application of stringent procedures and technological inventions [7]. Hand hygiene promotion and implementation of a bundle of targeted interventions for catheter-related infections are effective to prevent CRBSI [8-11]. It has been estimated that at least 56% of CRBSI could be prevented [12,13]. Guidelines typically include elements to assure sterile insertion and handling of central vascular catheters (CVC). These guidelines recommend less infection prone insertion sites, effective skin antisepsis, the use of specific catheter types, scheduled changes of device material and dressings, and formal training of involved healthcare workers with the use of checklists [14]. While dramatic successes in preventing CRBSI have been reported for more than a decade [8,9] guidelines are most likely not uniformly and successfully implemented in all European hospitals [15-17]. As another approach to reduce HAI, hand hygiene promotion has been associated with reduced incidence of bloodstream infection [18-20], but evidence from a randomized controlled trial is still lacking [21]. Hand hygiene performance remains suboptimal in most healthcare settings [22].

### Implementation of infection control best practices

It has now been widely accepted that infection control procedures be evidence-based. Yet, there is a large variability in how successfully hospitals implement programs and procedures to protect patients against HAI [23]. Only recently has more attention been devoted to finding out why wide variations exist in abilities of hospitals to translate evidence into practice [15-17]. Such variations in implementation success do not only depend on the quality of intervention programs but may likely be due to differences in the organizational context, which Øvretveit defines as ‘all factors that are not part of a quality improvement intervention itself’ [24]. Contextual factors are often cited as playing a role in the varied success of best practice interventions; however, existing literature reporting on implementation strategies often lacks details about specific contextual factors, and provides limited information about the real barriers and facilitators to implementation [25,26]. This is of particular importance given the present backdrop marked by a wide variety of cultures, healthcare organizations, and economies across European countries [27].

Thus, we are seeking to fill this gap in the field of infection control by following the call for further research by Greenhalgh *et al.* and answering the question: ‘By what processes are particular innovations in health service delivery and organization implemented and sustained (or not) in particular contexts and settings, and can these processes be enhanced?’ [28].

## Methods

### The PROHIBIT consortium study and its work packages

#### PROHIBIT consortium overall study design

This protocol is nested in a larger, consortium-led, European Union-funded study under the title ‘Prevention of Hospital Infections by Intervention and Training’ (PROHIBIT) [29], part of the European Commission 7^th^ Framework Program (FP7) [30]. The aim of PROHIBIT is: to understand existing guidelines and practices to prevent healthcare associated infections in European hospitals; to identify factors that enable and prevent compliance with best practices; and to test the effectiveness of interventions of known efficacy. Through its multiple work packages, PROHIBIT employs a mixed-methods approach, combining the strengths of a systematic review, quantitative surveys, qualitative research, and a randomized controlled intervention trial. Table 1 lists and briefly describes the six work packages of PROHIBIT.

**Table 1:** PROHIBIT work packages and their objectives

**Work package (WP)**	**Title**	**Objective**
WP 1	Project Management	Ensure that the project’s main scientific objectives are realized on schedule and on budget.
WP 2	Systematic review of European guidelines for HAI-prevention, surveillance and public HAI reporting	Detect and analyze current guidelines and recommendations in European countries for HAI-prevention of HAI. In addition, this work package will review HAI surveillance activities and schemes and public HAI reporting efforts in European countries.
WP 3	Survey of policy and practice for HAI-prevention in European hospitals	Assess the activity of European hospitals in HAI-prevention using a questionnaire of key determinants in a sample of hospitals in all European countries.
WP 4 (‘InDepth’)	In-depth qualitative investigation of success factors for adoption and implementation of infection prevention practices	Identify facilitators and barriers for successful adoption and implementation of evidence-based infection prevention practices by European hospitals.
WP 5	Randomized effectiveness trial of two interventions to reduce catheter-related blood stream infections	Demonstrate the effectiveness of implementation of two interventions to prevent CRBSI: 1) the WHO hand hygiene promotion strategy and 2) a CRBSI prevention bundle.
WP 6	Synthesis and dissemination	Provide tools for HAI-prevention to be used by stakeholders at multiple levels of health care systems.

CRBSI, catheter-related bloodstream infection; HAI, healthcare-associated infection(s); WHO, World Health Organization.

#### PROHIBIT work package 5: a randomized controlled intervention study

The PROHIBIT work package 4 (WP4), ‘InDepth’ follows the implementation of the CRBSI prevention initiative led by the PROHIBIT WP5 group out of Groningen, The Netherlands. WP5 tests the effect of two different approaches to CRBSI prevention (WHO hand hygiene promotion strategy and catheter bundle) in a three-arm, stepped wedged cluster-randomized controlled trial in a voluntary-based sample of 15 hospitals across Europe. The primary outcome for WP5 is the CRBSI rate. Secondary outcomes include compliance with hand hygiene and the CVC bundle.

The PROHIBIT WP5 organization and intervention strategies are described in Table 2. Intervention strategies 1, 2 or both have been sequentially rolled out in the 15 study hospitals over a period of at least 12 months, preceded by a baseline period of at least 6 months and an intervention period of 12 to 24 months.

**Table 2:** PROHIBIT work package 5 (WP5) organization and interventions

**Organization**	In each hospital, an onsite investigator has the primary responsibility for the local study-organization. Additionally, 0.5 full-time equivalent of a study nurse is paid by PROHIBIT who is responsible for performing CRBSI surveillance, measuring process outcomes, and implementing hand hygiene promotion and catheter care training, depending on which intervention package the hospital is randomized to. Study nurse received training in hand hygiene and CRBSI surveillance in Geneva, Switzerland.
**Intervention**	The study intervention includes focus group meetings with healthcare professionals of the participating hospitals (study nurses and intensive care physicians). Upon the meetings, the most recent evidence in CRBSI prevention and hand hygiene is delivered, and the participants will be trained in performing practical simulator training of catheter insertion and hand hygiene. Furthermore, a practical workshop on how to use a web-based e-learning tool for catheter care (http://www.carepractice.net) was organized, and they were provided with information about how to implement an intervention program successfully.
**Intervention strategy 1**	WHO hand hygiene promotion strategy based on materials designed by WHO and the University of Geneva Hospitals, Switzerland [31,32]. The five essential elements of the strategy are: 1) system change, including availability of alcohol-based hand rub at the point of patient care and/or access to a safe, continuous water supply and soap and towels; 2) training and education of health-care professionals; 3) monitoring of hand hygiene practices and performance feedback; 4) reminders in the workplace; 5) the creation of a hand hygiene safety culture with the participation of both individual healthcare workers and senior hospital managers.
**Intervention strategy 2**	CRBSI prevention bundle according to the Geneva model [33-35]: 1) hand hygiene; 2) maximal barrier precaution measures at CVC insertion (sterile gloves, cap, gown, large drape); 3) skin antisepsis with alcohol-based chlorhexidine; 4) subclavian access as the preferred insertion site; 5) early central line removal.

CRBSI, catheter-related bloodstream infection; CVC, central vascular catheter; WHO, World Health Organization.

#### PROHIBIT ‘InDepth’ work package: a qualitative inquiry

The present manuscript focuses on ‘InDepth.’ We pursue our qualitative inquiry in 6 of the 15 PROHIBIT WP5 study hospitals.

The “*InDepth*” qualitative inquiry asks two questions:

1. ‘Why are some hospitals/ICUs better at implementing CRBSI prevention than others?’

2. ‘What are the main barriers and facilitators to successful implementation of best practice in CRBSI-prevention?’

Taking advantage of quantitative results on the process and outcome level produced by WP5, the findings produced by ‘InDepth’ will ultimately be reported as a mixed-methods study.

#### Theoretical framework for the ‘InDepth’ inquiry

##### Implementation model: diffusion of innovation

Our model of the PROHIBIT WP5 implementation scenario follows a simplified scheme in a temporal stream based on the ‘diffusion of innovation’ paradigm [28,36]. Participating institutions would: 1) learn about PROHIBIT by invitation as members of the European Antimicrobial Resistance Surveillance System (EARSS); 2) make the decision to participate in PROHIBIT (adoption decision); 3) prepare the participation (appointing an onsite investigator, hiring a study nurse, informing ICU-leaders, building consensus); 4) start to monitor defined study outcomes (CRBSI-surveillance, hand hygiene observations); 5) launch their implementation process to align with the PROHIBIT protocol (re-writing of protocols, training of healthcare workers, evaluations, etc.); 6) institutionalize the intervention content. We are aware that, in reality, this process is likely to be much more iterative and complex.

#### Sensitizing schemes

Summarizing literature on diffusion of innovation [28] and implementation science [28,37] served as sensitizing schemes for this inquiry. Furthermore, we exposed ourselves to the theories of sensemaking [38] and new institutionalism [39]. For our purposes, we interpret new institutionalism as prolongation of sensemaking. According to this logic, individuals discovering something new will undergo a period during which they should make sense of it (name, categorize and relate it to concepts they are already familiar with) before it becomes part of the institution, aligning with social norms and expectations. The ‘Consolidated Framework for Implementation Research’ (CFIR) served us as a landscape constituted of numerous validated theories that facilitate translation of research findings into practice [37]. This framework also incorporates the extensive literature review and diffusion of innovation framework by Greenhalgh *et al.*[28].

#### Researchers’ influential backgrounds

Most of our research team members are healthcare workers and infection control specialists with an extensive past of leading promotional schemes and research. Thus, at the outset of the study, the team was familiar with elements of good infection control and patient safety activities, and the common challenges faced in achieving quality clinical practice.

#### Study definitions and procedures

##### Qualitative research

Study design and execution of ‘InDepth’ follows established criteria for qualitative research [40,41]. In the face of non-linear causality and complex interactions of adoption and implementation [42], qualitative research methods are particularly suitable to put quantitative findings into perspective, in particular when conceived as mixed-method studies [40,43-45]. The setting in this study is extremely complex and its elements interconnected given the variation in culture, healthcare systems, political and economic background, and infection control policies in Europe. This has been confirmed by unpublished preliminary results of PROHIBIT Work Packages 2 and 3. All of these contextual factors play an important role in the varied success of best practice interventions. In the context of implementation, such factors have been referred to as ‘conditions for improvement’ [24] and include elements that are both internal (*e.g.*, interpersonal relationships, safety culture, structural organizational characteristics) and external (*e.g.*, national regulations and financial incentives) to the implementing organization.

#### Case study method

We chose a case-study methodology with longitudinal follow-up to guide the qualitative investigation [46]. This approach is especially valuable for ‘InDepth’, as it allows for the investigation of the implementation phenomenon where the boundaries are not clearly defined between the context and the implementation itself. This may be intrinsically important to implementation success [47].

#### Case definition

The hospital is the unit of randomization considered for WP5, and consequently, we define each hospital as a primary case. Within each hospital case, we anticipate that individual nested case studies will emerge that detail the ICU-level or individual-level experiences, for example [46]. These nested, micro-case studies will serve to enrich the understanding of the overall primary cases.

#### Study hospital sampling strategy and procedure

Six hospitals were purposefully selected among the 15 WP5 intervention study hospitals based on the scheme of ‘extremes’ and ‘intensity’ [46,48]. Evaluation was performed by online questionnaire and telephone interviews with the onsite investigator to identify information-rich cases. While the questionnaire asks about concrete information like local healthcare, structure and organization of the institution and infection control, the interview confirmed questionnaire responses and provided more subtle information about the context of the institution. We identified six hospitals that would demonstrate the nature of the implementation process: three hospitals with a strong potential to succeed more easily, and three hospitals that would have to face more challenges to implement the PROHIBIT CRBSI and hand hygiene intervention.

#### Defining implementation success

Implementation success is defined and assessed on several levels in this study. Reduction of CRBSI will be the ultimate test for success, while high and sustained adherence to good practice will represent process quality. These measures will be produced under the WP5 protocol. To these numeric outcomes, we added a qualitative definition of successful implementation: the satisfaction of our interviewees with the intervention program, and the implementation process and outcome; the extent of ‘intervention fidelity’ (adherence to core content); and successful resolution of intervention-context fit issues (adaptation, local re-engineering, and innovation). Careful reflexivity and triangulation among the different sources of qualitative information will reflect the validity of this qualitative outcome measure [46].

#### On- and off-site data collection procedures

**Figure 1:**
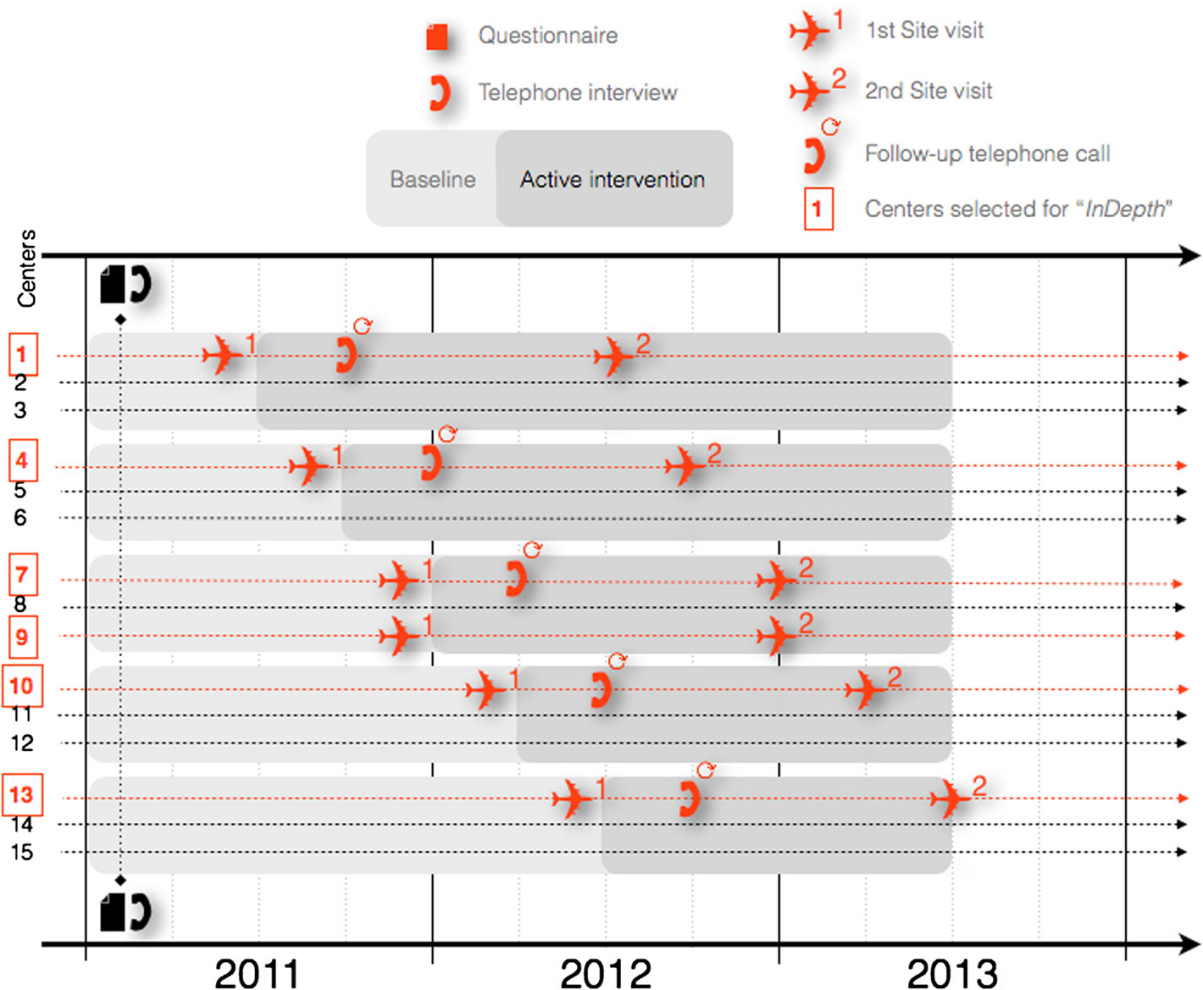
Temporal scheme of study procedures.

Onsite visits take place one month before and one year after the intervention launch (Figure 1). They include 10 to 15 interviews (Table 3) with key professionals from various levels and departments who were identified in advance or ad hoc (Table 4), and 2 to 3 ethnographic observations of 2 to 3 hours each in the ICUs focusing on ergonomic outlines, work attitude, hand hygiene behavior, catheter handling, and collection of written protocols. Three months into the implementation, additional telephone interviews with the onsite investigator were conducted to follow up on the implementation process. In addition, these follow-up telephone interviews and second site visits allow for member checking, *i.e.*, to verify our conclusions with study participants [46].

**Table 3:** A typical ‘InDepth’ interview guide

**Interviewee background**	Personal career?
	Role in the organization?
	Team environment and tasks?
**Biggest challenge**	What is your biggest challenge at work at this moment?
**Healthcare associated infections (HAI)**	Personal view of HAI?
	Perception of HAI by others in the institution?
**Prevention initiatives**	What were your past experiences with catheter-related bloodstream infections (CRBSI) or other HAI prevention, if any?
	(If no CRBSI experience, any other suitable initiative to discuss implementation was addressed)
	Probing for implementation dimensions*
**Adoption process**	How did the institution decide to participate?
**Safety culture**	Is leadership promoting patient safety?
	Do collaborators dare to speak up in case of unsafe behavior?
	How are critical incidents handled?
**Organizational culture**	Do you like working in this institution? What is great?
	Who is important?
	Staffing and other resources?

* Probing is based on dimensions and constructs from implementation frameworks [28,37].

**Table 4:** **List of typical *‘*InDepth*’ *criterion-selected interviewees**

**Top management level**	Chief executive officer (CEO)
	Director of nursing
	Medical director
**Infection control program**	Head of infection control program
	Infection control practitioner(s)/nurse(s)
	Infection control physician(s)
	Epidemiologist(s)
	PROHIBIT onsite investigator
	PROHIBIT onsite study nurse
**Intensive care unit (ICU) leaders**	ICU head physician
	ICU head nurse
**ICU frontline healthcare workers**	ICU nurse(s), specialized or in training
	ICU infection control link nurse
	Physician(s), specialized or in training

Staying for two days rather than just one allows us to take advantage of the exposure re-exposure effect [46] and to account for any Hawthorn effect. More details on the ‘InDepth’ data collection procedures can be found in Table 5.

**Table 5:** ***“InDepth” *data collection process**

**Identifying interviewees**	Sampling of candidates for interviews follows a ‘criterion strategy’ [46] in order to reach individuals at multiple levels of the institutional hierarchy and in key departments such as hospital administration, infection control program, and the intensive care unit (ICU). Additional interviewees are being selected following a ‘snowball sampling strategy’ based on recommendations of key informants by interview partners. Typical interviewees include individuals at top-management, ICU, and infection control level. See Table 3 for typical interviewee profiles. We planned for 10 to 15 interviews per site visit.
**Semi-structured interviews and interview guide**	Interviews are approximately one hour long and follow a semi-structured format, according to a ‘general interview guide approach’ [46]. Two researchers participate in each interview, with one person leading the conversation and the other backing up for recording, note-taking and occasional jump-in questions.
	Interview guides are prepared before each phase of the inquiry, listing questions and issues to be explored (Table 3). This approach ensures that the same lines of inquiry are explored, while still leaving freedom for each interviewee to elaborate on topics of particular interest. Interview sections and questions for the first series of site visits (before the intervention launch) were selected to understand background, position and network of the interviewee; the interviewee’s perception of healthcare-associated infection as well as those of other members of the organization; the experience of the institution with past implementation experience to estimate ‘implementation fitness’ and, more concretely, pre-existing achievements in catheter-related bloodstream infection prevention; rounding up with a broader view of safety and organizational culture.
	Interview guides for the second series of site visits (one year after the launch of the initiative) will be designed according to the cross-case analysis of the first series and in line with potential suggestions from the literature at that point in time.
**Ethnographic observations**	Ethnographic observations are useful as a tool for methodological triangulation. These direct, personal contacts, help us to better understand the context within which people interact, to obtain frank accounts from spontaneous, brief interviews, and to enrich data with a non-subjective view in agent behavior and sensemaking [49]. Two researchers perform observations at a time, taking notes when possible about the setting, activities taking place, who participates, and meaning of what was observed. Immediately after the observation session of 2 to 3 hours, observers individually produce narratives of the observation that serve as the primary material for the analysis alongside the interview transcripts. We perform 2 to 3 observation sessions per site visit.
**Photographic documentation**	Photography allows for documentation of rich, vivid accounts of the reality [46]. We take pictures of locations and important items, such as insertion sets and hand hygiene dispensers. Shots of the town and countryside further help to recall the context during the analysis. Additionally, we produce drawings of the unit layouts.
**Artifacts collection**	During site visits, we collect artifacts such as guidelines, written protocols, data collection and audit forms. Often, these have to be translated for the analysis.

#### Ethics approval

‘InDepth’ procedures were part of the document approved by the Institutional Review Boards of the 15 institutions participating in the PROHIBIT WP5 trial.

#### Analysis

We employ a ‘grounded approach’ to data analysis, which provides the opportunity to empirically build theory and identify new determinants and patterns rather than fit observations to existing frameworks and models [40,44,46]. This research process is iterative and emergent such that interim findings may influence the ongoing data collection. The case-level analysis is informed by Strauss and Corbin’s defined processes of grounded theory [50,51]. The conduct of the case-level analysis is detailed in Table 6.

**Table 6:** ‘InDepth’ case-level analysis scheme

**Onsite debriefings and cross-briefings**	Debriefings are conducted following each stage of data collection, during which the involved researchers note main observations and establish a tentative list of predicted barriers, facilitators, and emerging themes. This information is then cross-briefed with the remaining researchers while still on-site.
**Microanalysis, theme identification**	Following each visit, transcripts are read line-by-line by at least two researchers to identify recurring themes and to suggest relationships among them. These emerging themes are then discussed among all investigators to establish a list of main themes.
**Open and axial coding, theme memos**	Once the main themes for the site have been established, a process of open and axial coding [46] begins, during which all written data will be revisited and text will be coded in order to identify concepts and dimensions covered in the data. This coding is axial in that it identifies manifestations of each theme and its sub-themes at different levels of the organization, always occurring around the axis of the main theme. This analytical process results in the creation of theme memos, where each statement is supported by corresponding quotes or references to the observation narratives or artifacts and pictures.
**Case reports**	Once all theme memos are completed, they are discussed among all investigators to determine which themes are the most relevant to our central research questions and may have the most important implications on success or failure of the WP5 intervention. Relational ‘hypotheses’ are formulated about how concepts may relate in order to better understand phenomena taking place in the institution. All of this information is then compiled into a case report.
**Intermediate and final reports**	The above case-level analysis is completed for each of the six hospitals prior to and following the intervention. By combining these approaches and by using a sensitizing scheme (being aware of existing evidence of barriers and facilitators for diffusion of innovation and implementation in organizations) for the preparation and execution of the inquiry and using a grounded approach (generating theory directly from the material) for the analysis, a multidimensional picture of structural, organizational and interpersonal contexts will emerge as a basis to define local and universal barriers and facilitators for infection control interventions.

#### Reporting

Through this process of multilevel, qualitative and quantitative research, we seek to reveal the barriers and facilitators to implementation success, and to further identify which factors are recurrent across institutions [46]. As formative research, the information gathered from this inquiry will provide tools that can be used by stakeholders at multiple levels of healthcare systems, such as the EU, national policy makers, managers, and medical professionals, to guide future HAI-prevention initiatives.

#### Anticipated outcome of the study

We anticipate finding a set of universal and local barriers and facilitators for the implementation of infection control practices from the analysis of all cases. We predict them to be possible at any level of the institution, from single individuals, physical environment and tools, to formal and informal networks; processes inside the institution or directly linked to the intervention and the institutions’ and countries’ political, financial and cultural context. We also hypothesize that combinations of barriers and facilitators (rather than single most important determinants) will predict implementation success or failure.

#### Lessons learned so far

Study hospitals started according to the WP5 protocol with CRBSI surveillance and process measures in January 2011, and the first three study centers were randomized to launch their intervention in July 2011, followed by further three centers every quarter, the last three launching in July 2012 (Figure 1). Information and workshop days for study nurses were successfully conducted in Groningen, The Netherlands, and in Geneva, Switzerland. We completed our first series of before-launch site visits in six centers by June 2012. At this time, case study-level analysis has not been concluded, and cross-case synthesis has yet to start.

Centers are located in the following regions: Mediterranean, Baltic, British Isles, Alpine arc, and Visegard. We believe that the sample represents a balanced mix of different study intervention arms, geographical locations, and implementation success variation.

Three of the six included centers reside in regions outside of our group’s language fluency. We were, however, always able to organize the 10 to 15 interviews either in English or in the local language with the help of a local translator who was not part of the hospital or another authoritarian agency. We were welcomed in all hospitals and had no problems finding additional interviewees, mostly from frontline staff recruited during observations. The two days of presence in the field (including lunchtime and evenings in town) offered time for informal exchange with hospital contact people and/or the translator, which helped to enhance understanding of the wider context of these centers. In more than one instance, our stay was reported to have contributed to the dynamics of the PROHIBIT WP5 implementation process. In one instance, it was even seen as a breakthrough for the recognition of infection control by the institution’s leaders. This was unintended but not entirely avoidable, given our group’s background and affiliation with PROHIBIT. We tried to limit this by avoiding feedback or advice, even when solicited by centers. Moreover, we remained blinded to the quantitative outcome and performance results of PROHIBIT WP5 for each site throughout the inquiry.

## Discussion

This longitudinal case study is designed to provide contextual information to complement the results of the PROHIBIT intervention study by adding a qualitative, in-depth inquiry following the implementation of a CRBSI-prevention and hand hygiene promotion strategies in European ICUs. The results are expected to explain and to illustrate the epidemiological findings of the quantitative randomized controlled intervention trial. Moreover, they are intended to make the findings applicable in the sense of formative research.

The sensitizing exposure to theoretical frameworks of best practice implementation and the investigators’ professional experience in the field of infection control will ultimately help to relate our findings to the existing literature in both fields. Following existing schemes may be a barrier to discovering novel findings. We chose a grounded theory approach for data retrieval and analysis to counteract such limitations.

Overall, qualitative research methods are particularly effective to detect concepts and relationships in the context of high variability and interconnectedness of variables [46]. These contexts are highly applicable to understanding implementation processes. Thus, implementation research is urgently needed given the demand for unified global schemes to improve patient safety and healthcare effectiveness. Production of guidelines, even if based on published evidence, do not necessarily change healthcare worker practice [52]. This may be due to the lack of knowledge about barriers and facilitators inherent in local contexts and multidimensional patterns. Scientific knowledge on how to best address structural and organizational issues is scarce; even large-scale, well-funded patient safety initiatives may not guarantee success. A multisite quality of care improvement intervention in the United Kingdom based on the Institute of Healthcare Improvement (IHI) template channeling top management support, ‘walk-arounds’ with administrators, and the engagement of external change agents still failed to improve and sustain patient safety to a large extent [53,54]. The qualitative component of the cited study revealed enthusiastic top management buy-in at its commencement. However, enthusiasm failed to reach the healthcare workers at the bedside. Thus, we will pay special attention to the vertical alignment and frontline receptiveness of the PROHIBIT WP5 intervention.

### Protocols and scientific reports on qualitative studies of implementation of infection control best practice by others

Although understudied, there is some published qualitative research in the field of organizational factors of infection control best practice implementation. Uchida *et al.* used 23 semi-structured interviews in six acute care hospitals to gain insight in current practice in infection control under mandatory surveillance and reporting [55]. They found themes on mandatory reporting, technology on HAI surveillance, role expansion, and impact of organizational climate. An interdisciplinary group at the Veteran Affaires Hospitals and Michigan University in Ann Arbor has conducted several studies on the implementation of infection control best practice focusing on catheter-associated urinary tract infections [23,26,56,57]. They described the importance of champions in the implementation process and their dependency on working relationships within their organization [57]. It was considered ineffective to assign the role of champions, as these individuals are usually not intrinsically motivated.

Further, a published study protocol by Kyratsis and colleagues describes their qualitative research approach using in-depth comparative case study design to understand how healthcare managers draw upon sources of evidence, and how this translates into decisions about adoption and implementation of innovations in healthcare [58]. Via methods including in-depth semi-structured interviews, observations, and survey questionnaires in nine acute-care organizations, they aim to gain a broad understanding of organizational contextual dynamics, which could inform policy and decision makers responding to the present need for organizations to be innovation-ready.

### Why is this study unique?

Several features make this study a unique opportunity to learn more about the conditions under which implementation of best practices in infection control is likely to be successful. First, the diverse context of varying cultural, political and healthcare systems of the study cases will add robustness to the findings. Second, this background will be further understood as a result of simultaneous PROHIBIT work packages on national regulations and guidelines (WP2), and infection control practices (WP3; for both, see Table 1). Third, quantitative data for each hospital on CRBSI outcome, hand hygiene, and maximum barrier precautions adherence will allow for a state-of-the-art mixed-methods analysis [59].

A unique feature of our study methodology is that we follow the cases longitudinally with site visits before implementation and one year into the implementation of the PROHIBIT WP5 procedures. Just as a baseline measurement is essential in experimental study design, the site visits prior to implementation will be crucial in establishing a baseline estimate of the organization’s safety culture and implementation fitness and producing an inventory of CRBSI prevention measures already in place in the ‘InDepth’ study hospitals in order to identify what we call the ‘delta.’ This points to what CRBSI prevention elements the hospitals must actually implement or modify to achieve full PROHIBIT WP5 best practice implementation. Moreover, the second site visit allows for the detection of changes in the organization outside the PROHIBIT prevention initiative, since this implementation experience may trigger further changes towards best practice in other areas of the organization, the organizational structure, or even the safety culture in the sense of a halo effect.

### Limitations

#### Selection bias

There is some potential bias in the selection of the participating hospitals in PROHIBIT WP5. Hospitals first had to be part of the European Antimicrobial Resistance Surveillance System (EARSS) to be invited to participate in PROHIBIT; they had to be of large size to provide a sufficient number of central catheters; they had to have English-speaking professionals to serve as onsite investigators; and they had to have an ICU. Furthermore, the hospitals had to agree to send physicians and nurses abroad for training. These are all factors that could bias our study towards hospitals more receptive to change. However, we believe that the barriers and facilitators to implementation will fundamentally apply to more challenged institutions, even so to a larger extent.

#### Potential bias in researchers

Two study group members were occasionally involved in the training of ICU physicians and nurses in the centralized workshops of PROHIBIT WP5. Here, they gained detailed information regarding participating hospitals and interacted with HCWs whom they would again see upon one of the site visits. Obtaining information before the site visit may contribute bias, but is also a source of useful information. Heightened attention to reflexivity serves as a safeguard against this type of bias [60].

## Conclusions and outlook

With its longitudinal and mixed-method research design among a variety of healthcare contexts across a multicultural Europe, this study will provide actionable information about barriers and facilitators to the implementation of best practices in infection control. The multicultural background and numerous logistic and communication issues do pose some challenges for this research. After having completed the first round of site visits, however, we have gained firm confidence in its feasibility and potential for success. We anticipate that, some years later, these same cases could become the subject of further investigation into the question of sustainability.

### Consent

Written informed consent was obtained from all informants for publication of this report.

## Competing interests

The authors declare that they have no competing interests.

## Authors’ contributions

HS conceived of the study, participated in its design and in data collection, and drafted and finalized the manuscript. LC participated in data collection, protocol design, and substantially contributed to writing the manuscript. ST participated in data collection and protocol design. FLJ participated in data collection, protocol design, and was responsible for project management. DP participated in the conception of the study protocol and writing of the manuscript. WZ participated in study design revisions, data collection, and manuscript writing. All authors read and approved the final manuscript.
